# Skeletomuscular adaptations of head and legs of *Melissotarsus* ants for tunnelling through living wood

**DOI:** 10.1186/s12983-018-0277-6

**Published:** 2018-08-14

**Authors:** Adam Khalife, Roberto A. Keller, Johan Billen, Francisco Hita Garcia, Evan P. Economo, Christian Peeters

**Affiliations:** 1 Sorbonne Université, CNRS, Institut d’Écologie et des Sciences de l’Environnement, 75005 Paris, France; 2 Biodiversity and Biocomplexity Unit, Okinawa Institute of Science and Technology Graduate University, Onna, Okinawa, Japan; 3 MUHNAC/cE3c: Centre for Ecology, Evolution and Environmental Changes, Faculdade de Ciências, Universidade de Lisboa, Lisbon, Portugal; 4 Laboratory of Socioecology and Social Evolution, Zoological Institute, K.U. Leuven, Belgium

**Keywords:** Micro-CT, Formicidae, Mandibles, Apodemes, Diaspidids, Mutualism, Biomechanics, Adaptation

## Abstract

**Background:**

While thousands of ant species are arboreal, very few are able to chew and tunnel through living wood. Ants of the genus *Melissotarsus* (subfamily Myrmicinae) inhabit tunnel systems excavated under the bark of living trees, where they keep large numbers of symbiotic armoured scale insects (family Diaspididae). Construction of these tunnels by chewing through healthy wood requires tremendous power, but the adaptations that give *Melissotarsus* these abilities are unclear. Here, we investigate the morphology of the musculoskeletal system of *Melissotarsus* using histology, scanning electron microscopy, X-ray spectrometry, X-ray microcomputed tomography (micro-CT), and 3D modelling.

**Results:**

Both the head and legs of *Melissotarsus* workers contain novel skeletomuscular adaptations to increase their ability to tunnel through living wood. The head is greatly enlarged dorsoventrally, with large mandibular closer muscles occupying most of the dorsal half of the head cavity, while ventrally-located opener muscles are also exceptionally large. This differs from the strong closing: opening asymmetry typical of most mandibulated animals, where closing the mandibles requires more force than opening. Furthermore, the mandibles are short and cone-shaped with a wide articulatory base that concentrates the force generated by the muscles towards the tips. The increased distance between the axis of mandibular rotation and the points of muscle insertion provides a mechanical advantage that amplifies the force from the closer and opener muscles. We suggest that the uncommonly strong opening action is required to move away crushed plant tissues during tunnelling and allow a steady forward motion. X-ray spectrometry showed that the tip of the mandibles is reinforced with zinc. Workers in this genus have aberrant legs, including mid- and hindlegs with hypertrophied coxae and stout basitarsi equipped with peg-like setae, and midleg femura pointed upward and close to the body. This unusual design famously prevents them from standing and walking on a normal two-dimensional surface. We reinterpret these unique traits as modifications to brace the body during tunnelling rather than locomotion per se.

**Conclusions:**

*Melissotarsus* represents an extraordinary case study of how the adaptation to – and indeed engineering of – a novel ecological niche can lead to the evolutionary redesign of core biomechanical systems.

**Electronic supplementary material:**

The online version of this article (10.1186/s12983-018-0277-6) contains supplementary material, which is available to authorized users.

## Background

Social insects can shape their living environment dramatically through the coordinated efforts of nestmates working in unison over time. A wide range of ants (as well as termites) are known to construct extensive networks of underground tunnels and chambers. Within these, some ant species host symbionts such as cultivated fungi or sap-sucking insects that provide honeydew rewards [1, 2]. Such long-lived nests are excavated in soil using the mandibles. Arboreal nests seldom reach the same physical scales, except when host plants provide pre-existing living spaces. In other cases, tunnels are usually carved in dead tissues of standing or fallen trees, with only a minority of ant species able to chew through healthy living wood. The latter generally involve chewing an entrance hole or short tunnel to a pre-existing cavity. While many arboreal ants obtain trophic benefits from scale insects, only relatively few species keep their partners inside the nest chambers.

**Fig. 1:**
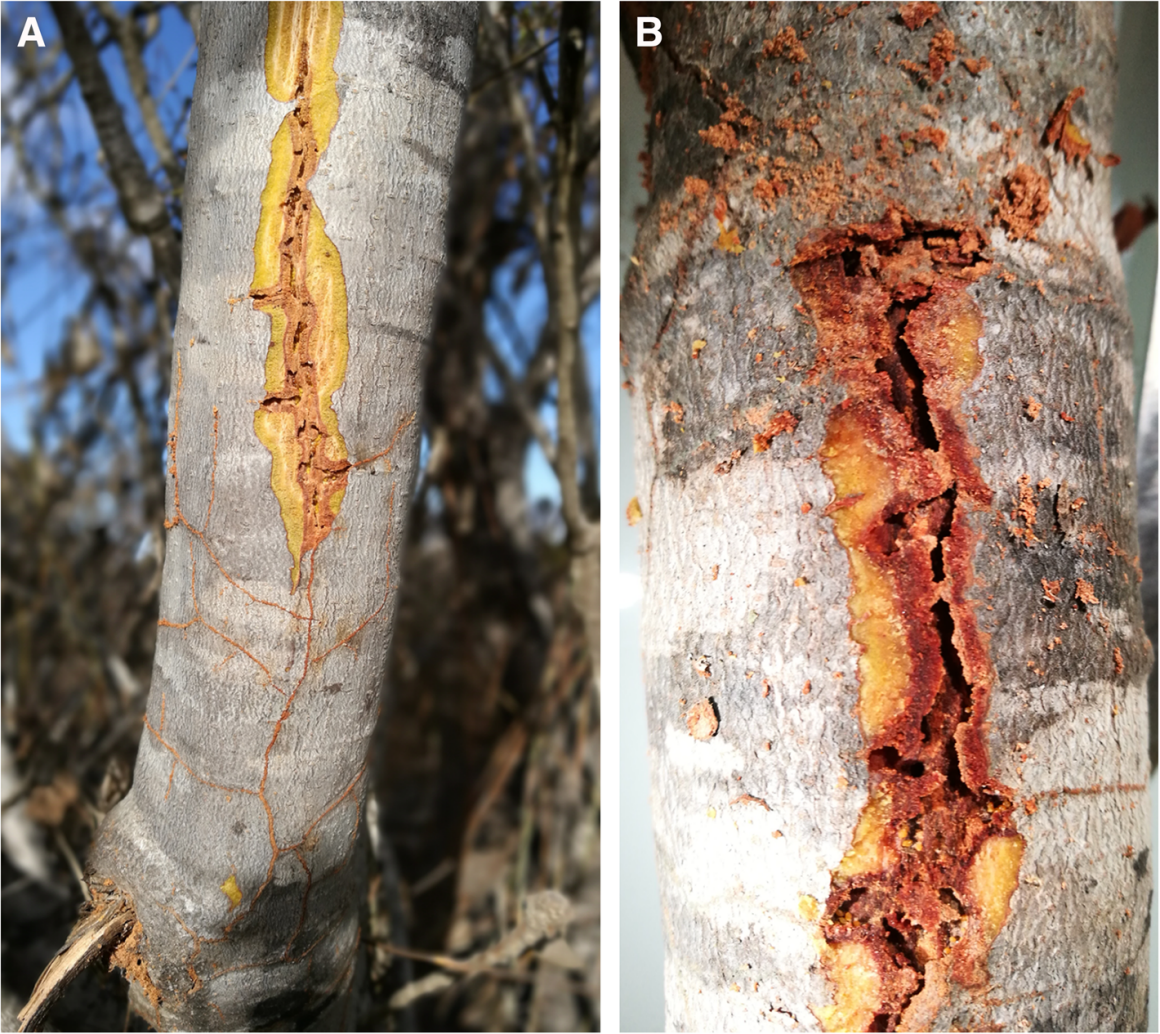
*Melissotarsus*-infested *Leucospermum praemorsum* (Proteaceae) trees in Western Cape, South Africa. Sections of the bark were scratched open with a knife, revealing the extensive network of tunnels. **a** Outline of tunnels is visible with the bark intact (bottom of image). **b** Close-up of tree trunk

**Fig. 2:**
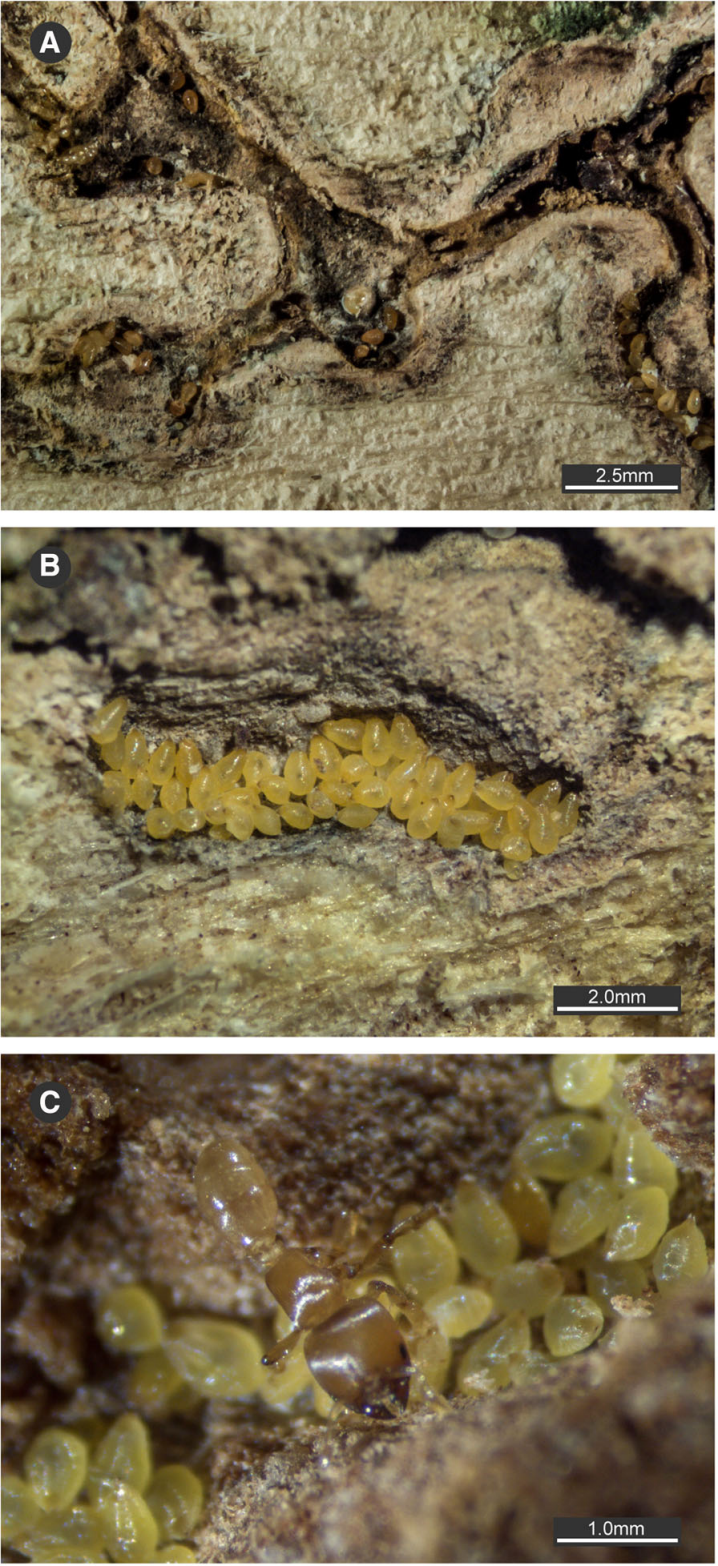
*Melissotarsus* workers and associated scale insects in *Olax dissitiflora* trees. **a** Local network of tunnels under the bark. **b** Aggregate of diaspidids. **c** A worker with diaspidids


*Melissotarsus* (subfamily Myrmicinae) are anomalous among arboreal ants: these minute ants (2–2.5 mm long) live exclusively under the bark of living trees throughout Africa and Madagascar (Fig. 1), chewing large networks of tunnels that are inhabited by many thousands of armoured scale insects (family Diaspididae) (Fig. 2) [3]. The adaptations that facilitate this lifestyle are not fully understood, even though *Melissotarsus* has a morphology unique among ants. The head of workers is dorsally enlarged and lateroposteriorly broad as in various other large-headed ants. However, the head capsule of *Melissotarsus* is not bilobed, but rather expands dorsoposteriorly into a single broad lobe that overhangs the connection between head and neck (occipital foramen). Another outstanding trait of this genus is a pronounced ventral expansion of the head cavity forming a concave internal floor. But the most obviously unusual feature of *Melissotarsus* workers is their aberrant legs. The first pair has expanded basitarsi bearing thick brushes of setae to spin silk [4, 5]; the second and third pairs have huge coxae and thick basitarsi armed with peg-like setae at their distal end; all three pairs of legs have weak distal tarsomeres. Moreover, the femur of the midlegs is directed upward and workers are unable to walk outside their tunnels [6] (see Additional file 1). Hence all their food must be obtained within the tree: wax and proteins secreted by the diaspidids to build their shields (scale insects in this family excrete no honeydew), as well as live individuals and exuviae [3]. The tunnelling prowess of *Melissotarsus* workers provides their diaspidid partners with a safe environment under the bark, close to the parenchyma tissues they feed on. However, the function of their aberrant legs is not clear and was investigated further.

Additional file 1:Video of walking attempts of a *Melissotarsus* sp. worker taken out of the tunnels of its nest inside a living tree (Nardouwsberg, Western Cape, South Africa). (MP4 10095 kb)


It is likely that the *Melissotarsus* head is specialized for chewing. The largest muscles within the mandibulated insect head are those that operate the mandibles [7]. Each mandible is controlled by one set of “closer” and one set of “opener” muscles. These muscles act antagonistically by pulling opposite extremes of the mandibular base, causing mandibles to swing towards or away from the mouth (closing and opening motions respectively). These muscles have been investigated in various ants that use mandibles to hunt. In particular, trap-jaw ants have mandibular muscles that generate phenomenal closing speeds, either by direct action or the use of power amplification mechanisms (reviewed in [8]). However, much less attention has been given to ants having mandibles designed for sustained strength, e.g. cracking seeds, biting enemies, dismembering prey, and tunnelling. Wood-boring insects, including larvae and/or adults of many Coleoptera, Lepidoptera, Isoptera and Hymenoptera (e.g. woodwasps, carpenter ants), usually tunnel through dead or decayed wood [9, 10], or chew very short galleries to reach the center (pith) of living branches (e.g. *Gesomyrmex* ants) [11]. Compared to decayed dry wood, chewing healthy wood requires more strength because moisture keeps wood fibres elastic as opposed to brittle [12]. *Melissotarsus* nests apparently extend throughout entire trees [13]. In social insects, large nests are attributed to division of labour involving numerous participants, but the superior morphological characteristics of individual participants also need proper emphasis.

In this study, we used complementary data from serial histological sections, SEM imaging, micro-CT 3D reconstruction, and X-ray (EDX) spectrometry, to test the hypothesis that the *Melissotarsus* mandible and leg systems have been modified for wood-tunnelling. We reinterpret the characteristic leg morphology of *Melissotarsus* workers as specialization for anchoring the body during tunnelling, to resist powerful backward forces exerted by the mandibles.

## Methods

### Study organisms

Colonies of *Melissotarsus* spp. containing many workers, queens, brood and diaspidids were sampled in Gorongosa National Park, Mozambique (August 2016) and Nardouwsberg, Western Cape, South Africa (May 2017). Inhabited branches were transported to Paris. Extremities of the branches were kept moist, allowing to keep ants and diaspidids alive for several weeks.

Despite the compelling morphological distinctiveness of the genus *Melissotarsus* as a whole, species-level taxonomy is confusing and challenging given the high degree of conservatism in worker morphology across Africa and Madagascar. The genus was revised by Bolton (1982) who recognised three species in Africa [14]. However, given that specimens were available from only few localities, Bolton noted that species boundaries are difficult to ascertain: there is either one widely distributed species, or more than three species. Without a modern taxonomic revision, it is currently impossible to determine the genuine species identity of the material used in this study. Consequently, unique identifiers are given for all specimens scanned (see Additional file 2). Voucher specimens are held at OIST. We refrain from applying any species name and use *Melissotarsus* instead, emphasizing that all the morphological features studied here apply to the genus as a whole.

*Messor barbarus* workers were collected in Montpellier (Southern France) and Lisbon (Portugal), and used as a morphologically unspecialized Myrmicinae for comparison.

### Histology

*Melissotarsus* worker heads were fixed in cold 2% glutaraldehyde buffered at pH 7.3 with 50 mM sodium cacodylate and 150 mM saccharose. Postfixation was done in 2% osmium tetroxide in the same buffer. Dehydration was achieved in a graded acetone series, and tissues were embedded in Araldite. Serial sections of 1 μm were made with a Leica EM UC6 ultramicrotome, then stained in methylene blue and thionin and viewed with an Olympus BX-51 microscope. Three heads provided 630 longitudinal, 610 transversal and 400 frontal sections.

### X-ray spectrometry

To check for the eventual presence of zinc in the mandibular tip, we analyzed double stained thin sections (prepared as described above) in a Jeol ARM-200F electron microscope equipped with a probe aberration corrector, operated at 200 kV. The microscope is equipped with an energy dispersive X-ray (EDX) spectrometer to perform EDX measurements at a collection angle of 0.98 sr and with a 100 mm^2^ detection area.

### Sarcomere length measurement

On the frontal sections, some closer muscle fibres were half-relaxed and half-stretched, due to slow penetration of the fixative (W. Gronenberg, personal communication). Sarcomeres were measured in both the relaxed and stretched parts of 15 fibres, allowing us to infer that closer fibres were relaxed on the transversal and most of the frontal sections. Opener fibre sarcomeres, only measurable on the frontal sections, were all stretched: measured lengths were multiplied by the empirical ratio relaxed/stretched calculated from the half-relaxed half-stretched closer fibres. To get accurate measurements, five sarcomeres at a time were measured on several fibres of various regions of the head, yielding a single sarcomere length value per fibre.

### Scanning Electron microscopy

Whole unpinned workers fixed in 96% ethanol were air-dried and point-mounted in a natural position. Mounted specimens were coated with gold-palladium and imaged using a Hitachi S4700 field emission scanning electron microscope at a voltage of 5–10 kV.

### X-ray micro-computed tomography

Micro-CT scans were performed using a Zeiss Xradia 510 Versa 3D X-ray microscope operated with the Zeiss Scout-and-Scan Control System software (version 11.1.6411.17883) at the Okinawa Institute of Science and Technology Graduate University, Japan. Material of *Melissotarsus* sp. and *Messor barbarus* was initially preserved and stored in 90% ethanol. Prior to the scanning procedure the specimens were stained in a 2 M iodine solution for 24 h, and subsequently transferred into microtubes filled with 99% ethanol. Scan settings were selected accordingly to yield optimum scan quality. Full 360 degree rotations were based on 1601 projections. The resulting scans have resolutions of 990 × 1013 × 988 pixels and an overview of the specimens used and scanning parameter settings is provided (see Additional file 2: Table S1). Post-imaging 3D reconstruction was done with the Zeiss Scout-and-Scan Control System Reconstructor software (version 11.1.6411.17883), and the output files saved in DICOM format.

### 3D modelling

3D reconstruction of image stacks was first visualized with Drishti 2.6.3 [15], a software that only uses voxel intensity to build 3D models. Transfer Function Editor, Point/String Light, Clipping Plane and Viewport tools were used for internal and external visualization of the worker and queen head, mesosoma and legs. Then, active voxel designation (i.e. segmentation) of the reconstructed image stacks was performed with ITK-SNAP 3.6.0 [16] for one half of the *Melissotarsus* worker head. The ‘region competition’ algorithm was used for 3D automatic segmentation, which was followed by manual segmentation to correct the boundaries of some structures, for example between the cuticle and the muscles involved in mandible closing. Muscles and skeletal structures were annotated by homology relative to *Apis mellifera* following Snodgrass (1956) [17]. The resulting segmentation was exported as vtk mesh files. A transparent rendering of the full head was created with the Isosurface tool in Amira software (version 6.3.0), and then exported as a ply mesh file. Meshes were opened in ParaView (version 5.4.1) for visualization, snapshots, and animation. Mandible, apodemes and mandible muscles were segmented similarly for one half of the head of a *Messor barbarus* worker, then analysed with the same workflow.

### Direct dissections

To complement the information from virtual 3D models, we dissected worker heads. To assess musculature, heads fixed in 80% ethanol were cut open with a razor blade at various angles. For the assessment of cuticular skeleton, individuals were put in a 12% potassium hydroxide (KOH) solution overnight to remove all the soft tissues. These preparations were examined under a Wild M5 stereomicroscope.

## Results

### External morphology

**Fig. 3:**
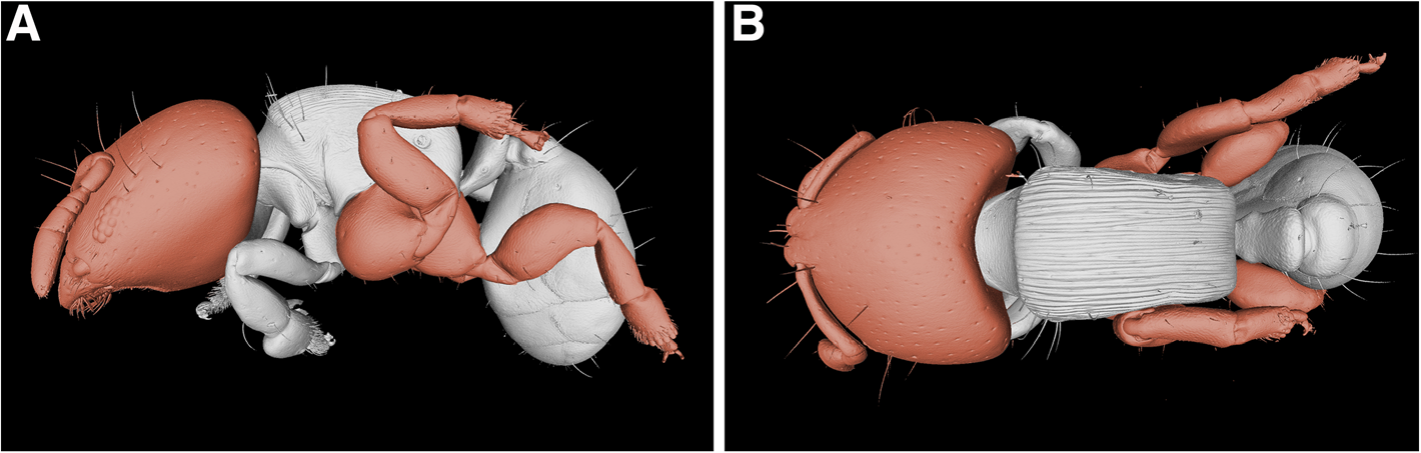
**a** Lateral and **b** dorsal views of a *Melissotarsus* worker (CASENT0790873). The relatively large head, hypertrophied coxae, large basitarsi, and the peculiar orientation of the midlegs are highlighted in red

**Fig. 4:**
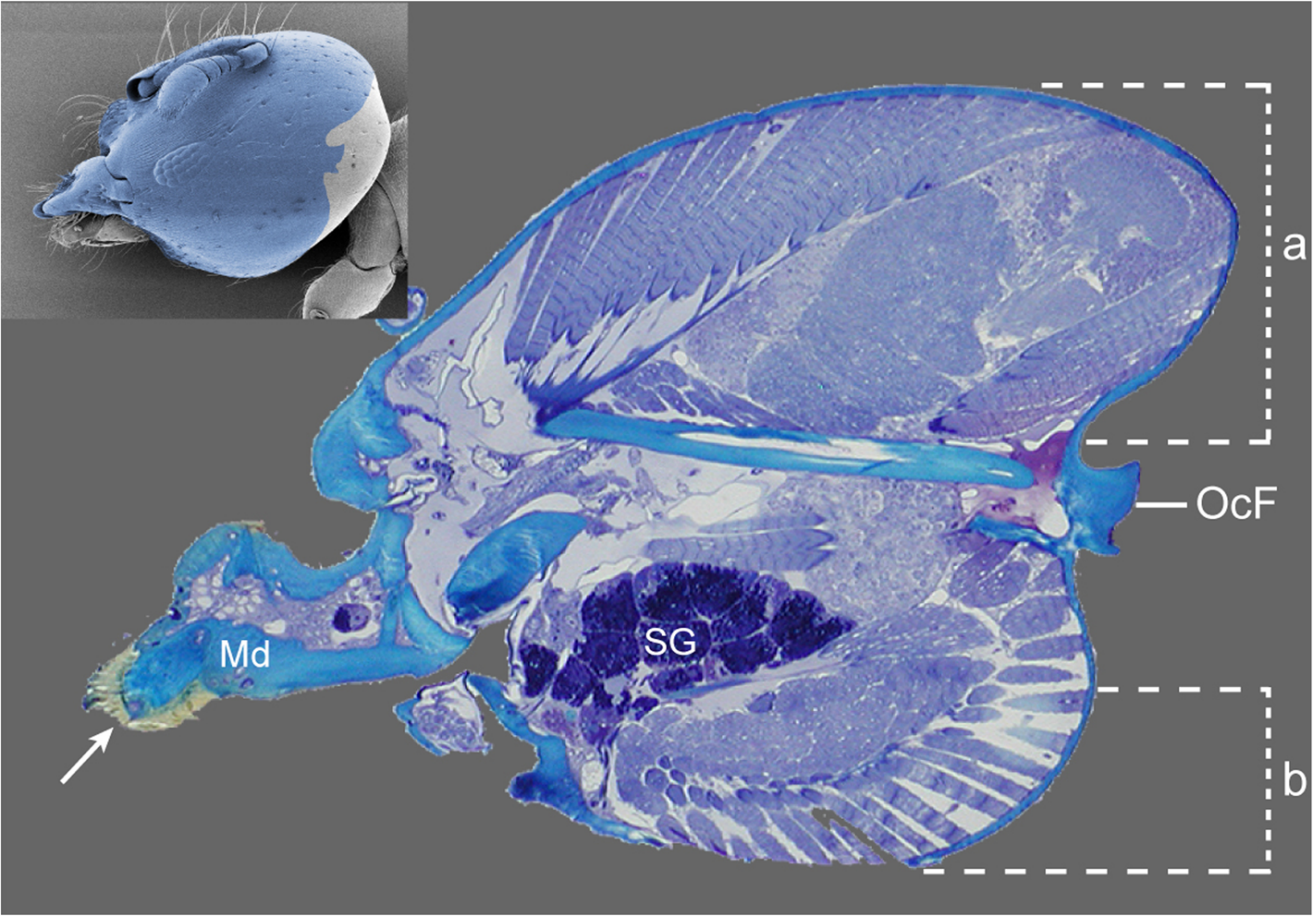
Sagittal histological section of the head of a *Melissotarsus* worker. This shows the extent of (**a**) dorsoposterior lobe and (**b**) ventral expansion. Arrow points at the zinc reinforcement on the mandible tip. Inset shows SEM of head with area of the histological section indicated in blue. Md, mandible; OcF, occiput foramen; SG, silk gland

*Melissotarsus* workers are compact large-headed individuals, with relatively short but stout legs (Fig. 3D model [18]). The middle and hind pairs have hypertrophied coxae and heavy basitarsi equipped with peg-like setae (see Adaptations of mid and hind legs). The relative size of the head of workers is comparable to what is found in majors and soldiers of various ant species, even though the more robust thorax in *Melissotarsus* makes this less conspicuous. The head capsule of workers expands dorsoposteriorly and ventrally, increasing the overall volume (Fig. 4, top-left corner). Our 3D reconstructions [18] and SEM imaging showed that the head of queens is normal in shape and lacks the dorsoventral modifications of workers.

### Mandible muscles and apodemes

**Fig. 5:**
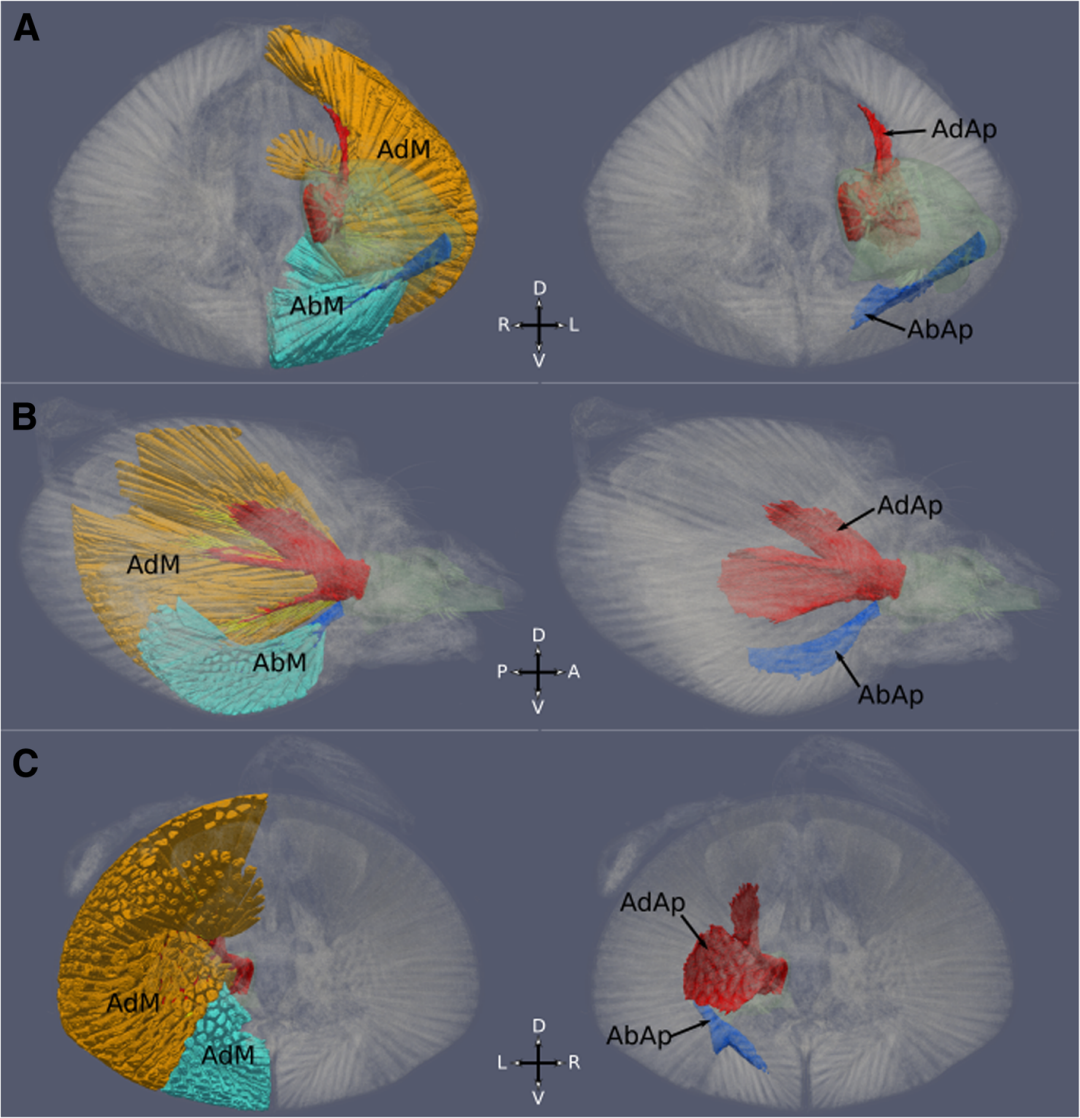
3D reconstruction and segmentation of the mandible muscles and apodemes of a *Melissotarsus* worker. **a** Anterior, **b** lateral, and **c** posterior views; (left) muscles and apodemes, (right) apodemes only. AdM, closer muscle (orange); AbM, opener muscle (light blue); AdAp, closer apodeme (red); AbAp, opener apodeme (dark blue)

3D segmentation showed that most of the head cavity is occupied by huge mandible muscles for closing (adduction) and also for opening (abduction) (Fig. 5). Closer muscle fibres originate along most of the dorsal roof, sides, and the dorsoposterior lobe of the head, and insert into a large mandible closer apodeme. This closer apodeme follows the basic design observed in other ants [19]: highly sclerotized and consisting of a main central process from which two subprocesses branch out on each side (Fig. 5). The main process is a large blade oriented vertically and parallel to the side of the head, catching most of the muscle fibres that originate laterally and posteriorly (video of surface rendering of internal head structures [20]). The lateral branch is also large and blade-like, more dorsally oriented, and receives the numerous muscle fibres originating at the dorsal enlargement of the head. Finally, the medial branch is an elongated spatula for the insertion of muscle fibres originating within the posterior lobe that overhangs the occipital foramen.

The opener muscles are considerably enlarged (4.6% of the head volume compared to 1.1% in *Messor barbarus*). They fill the ventral enlargement of the head, with fibres originating along its concave floor, posteroventral wall of the head, and the large ventromedial phragma that runs longitudinally across the floor of the enlarged ventral cavity. Unlike what is observed in other ants, we found large sclerotized opener apodemes, each consisting of a single broad blade-like process oriented horizontally and parallel to the floor of the head (Fig. 5). Like the closer apodemes, these opener apodemes provide a large surface area for the insertion of the unusually large opener muscles.

The brain is a large central organ whose position conflicts with mandible muscle fibres that cross the head anteroposteriorly. More precisely, the lateral optic lobes are a direct constraint on the volume of the closer muscles and the geometry of the closer apodeme. In *Melissotarsus* workers, optic lobes are reduced along with eye size. In addition, space between the brain and the muscles is minimal, with fibres passing one micron away over and under the optic lobes. Importantly, the *Melissotarsus* head also contains multiple clusters of hypostomal silk glands (Fig. 4), as well as pheromone-producing intramandibular and mandibular glands, enzyme-producing propharyngeal glands, and lipid-metabolizing postpharyngeal glands. These glands are additional constraints on the geometry and volume of the mandibular apparatus.

For both closer and opener muscles we distinguished direct fibres inserting on the apodemes, as well as indirect fibres connecting the apodemes via a membranous filament. Closer direct fibres had longer sarcomeres than closer indirect fibres (6.6 ± 1.0 μm vs 5.6 ± 0.6 μm, Wilcoxon test, *p* < 0.001). Direct fibre sarcomeres were also wider than indirect fibre sarcomeres for the opener muscles (8.4 ± 0.6 μm vs 7.4 ± 0.5 μm, Wilcoxon test, *p* < 0.01). Opener muscle sarcomeres were longer in both direct and indirect fibres (Wilcoxon test, *p* < 0.001 for both).

### Mandible shape and articulation

**Fig. 6:**
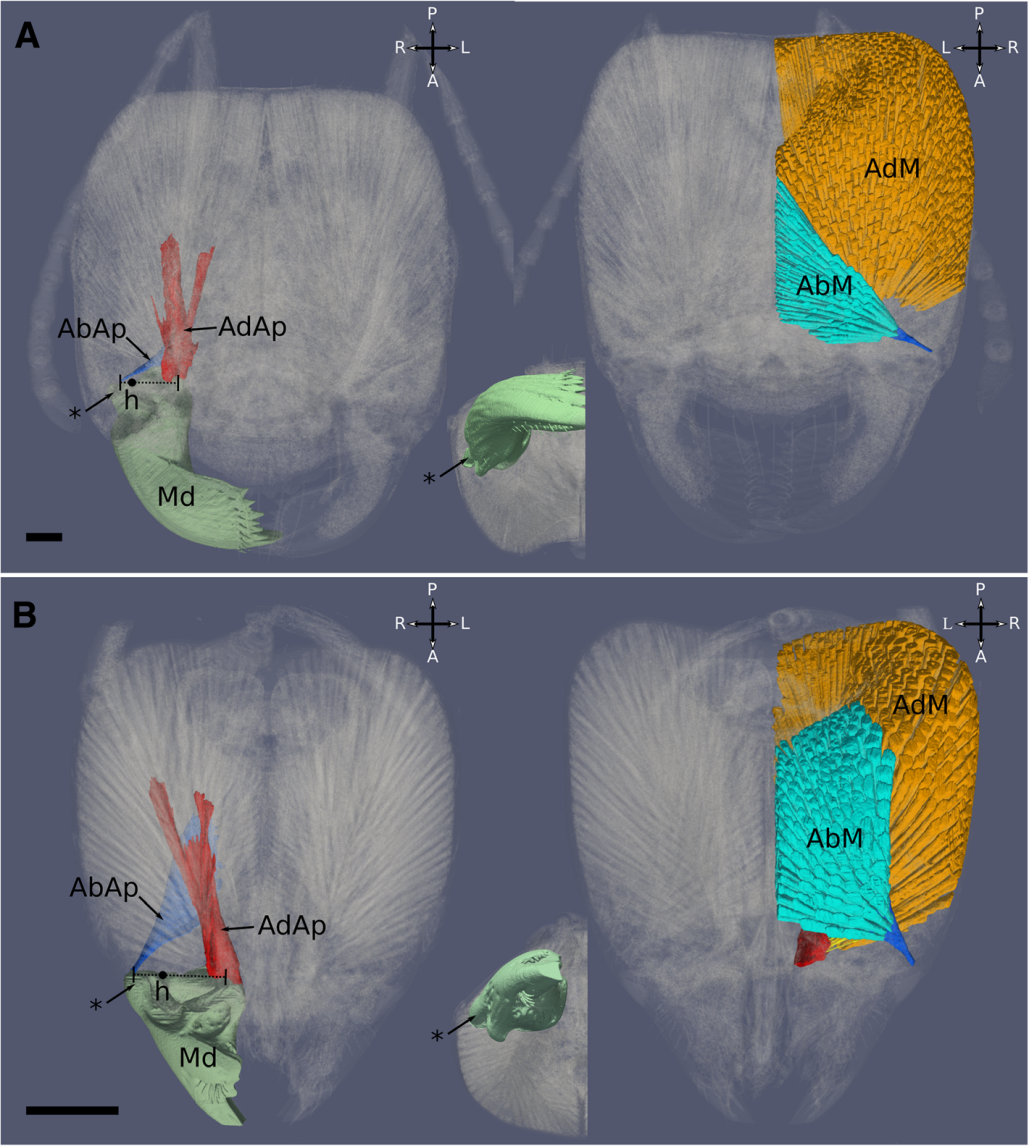
3D reconstruction and segmentation of mandible with apodemes and muscles of *Messor* and *Melissotarsus* workers. Left: apodemes only (dorsal view). Right: muscles and apodemes (ventral view). Inset: mandible with lateral process (anteroventral view). **a** *Messor barbarus.* **b** *Melissotarsus.* AdM, closer muscle (orange); AbM, opener muscle (light blue); AdAp, closer apodeme (red); AbAp, opener apodeme (dark blue); Md, mandible; h, hinge (point of mandible rotation); *, mandible lateral process. Scale bar: 0.2 mm

**Fig. 7:**
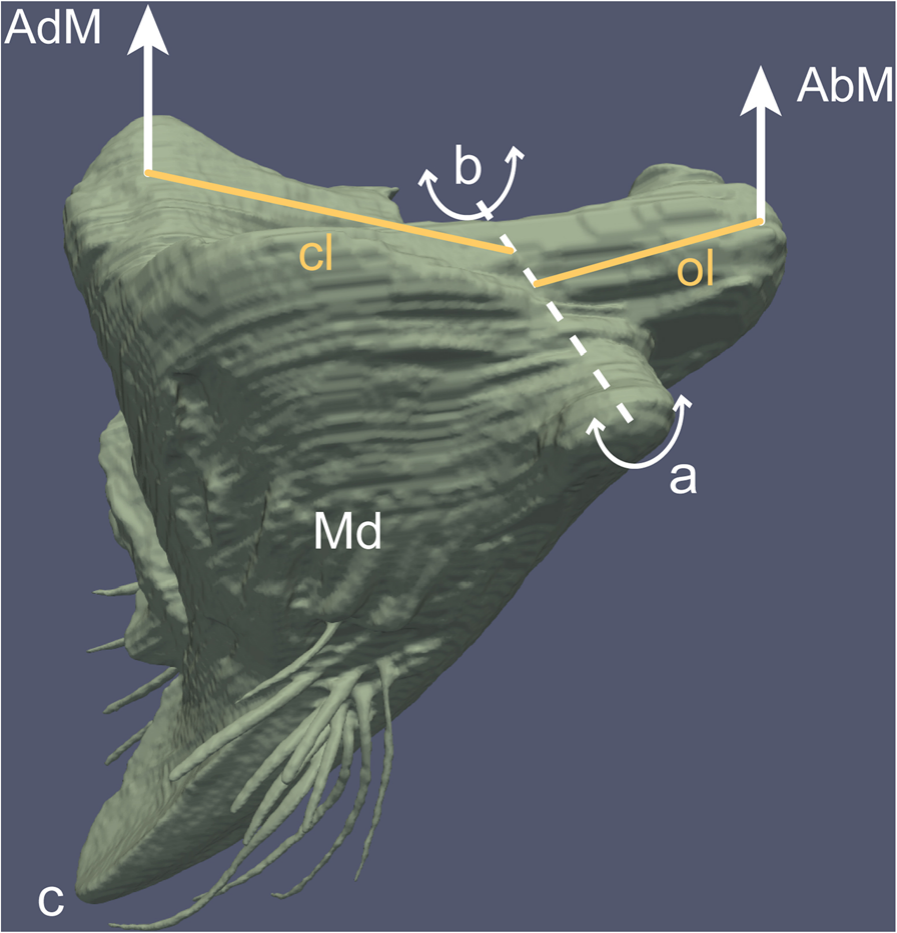
Mandible shape and lever effects in *Melissotarsus* worker. AdM, closer muscle insertion; AbM, opener muscle insertion; Md, mandible: a, ventral condyle; b, dorsal acetabulum; dashed line, mandible axis of rotation (hinge); c, mandible tip; cl, closer lever; ol, opener lever

The mandibles of *Melissotarsus* workers are short and cone-shaped with a base that is as broad as their length. This contrasts with the unspecialized mandibles of *Messor* workers, with a narrow base and a long stem from which a thin triangular blade projects medially (Fig. 6). Notably, the lateral process of each mandible where the opener muscle attaches is particularly long, increasing the distance between the mandibular hinge and the insertion of this muscle. The peculiar large broad base of *Melissotarsus* mandibles is important because an increased distance between the axis of mandibular rotation and the points of muscle attachment gives a mechanical advantage that amplifies any force generated by the already large closer and opening muscles (Fig. 7).

**Fig. 8:**
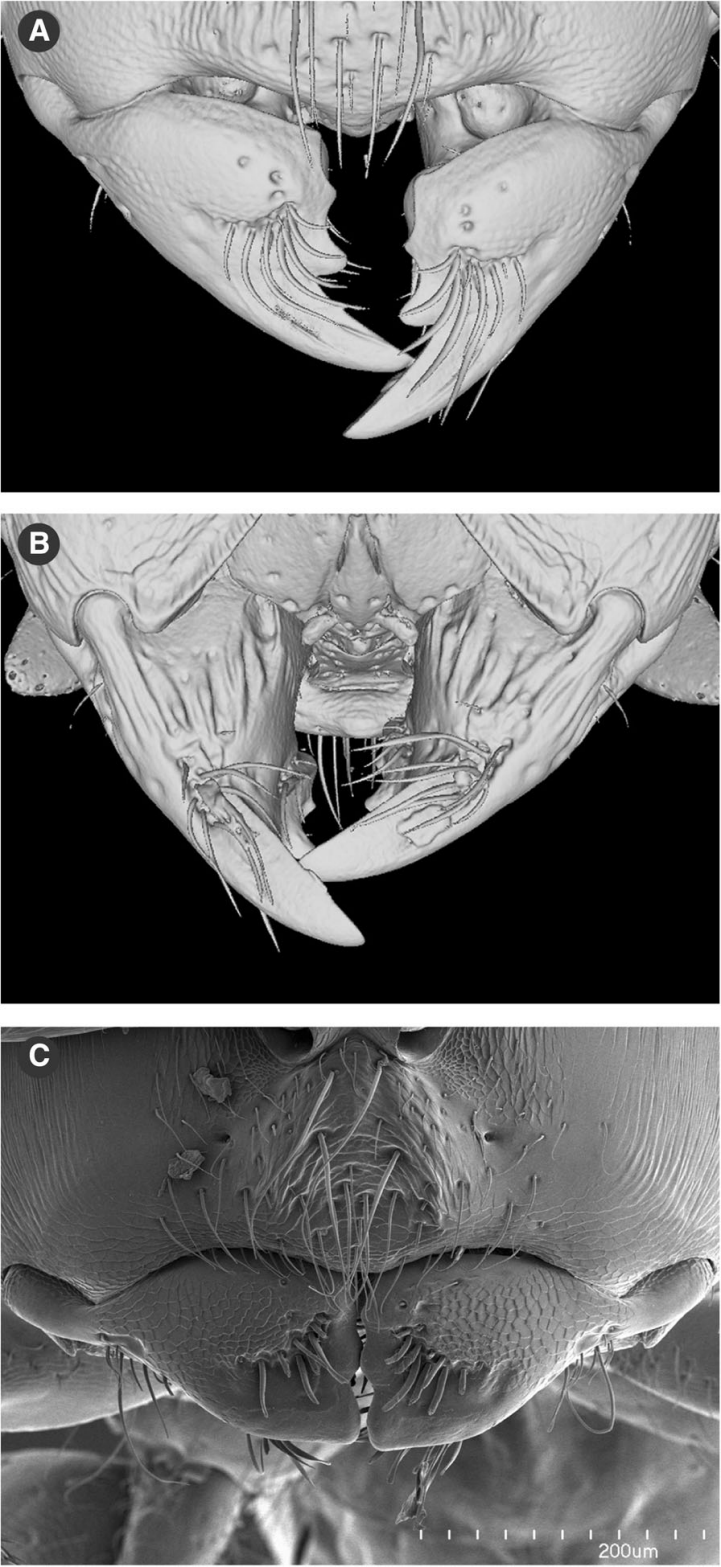
Mandibles of *Melissotarsus* worker. 3D surface model in **a** dorsal and **b** ventral view of the mandibles of a young worker. **c** SEM image in dorsal view of the blunt mandibles of an old worker

Young workers of *Melissotarsus* can be identified by the acute pointed tips of their mandibles, which wear out and become blunt after constant use (Fig. 8c). Histology suggested the presence of zinc in the mandibular tips (Fig. 2), and this was confirmed by TEM-EDX spectrometry (no zinc could be detected in the occiput that was checked as a control). Turfs of long setae occur midway on both dorsal and ventral surfaces of mandibles, directed towards the tip (Fig. 8a, b). These setae never exceed the tip of unworn mandibles but will also become shorter and blunt as workers age (Fig. 8c).

### Adaptations of mid and hind legs

**Fig. 9:**
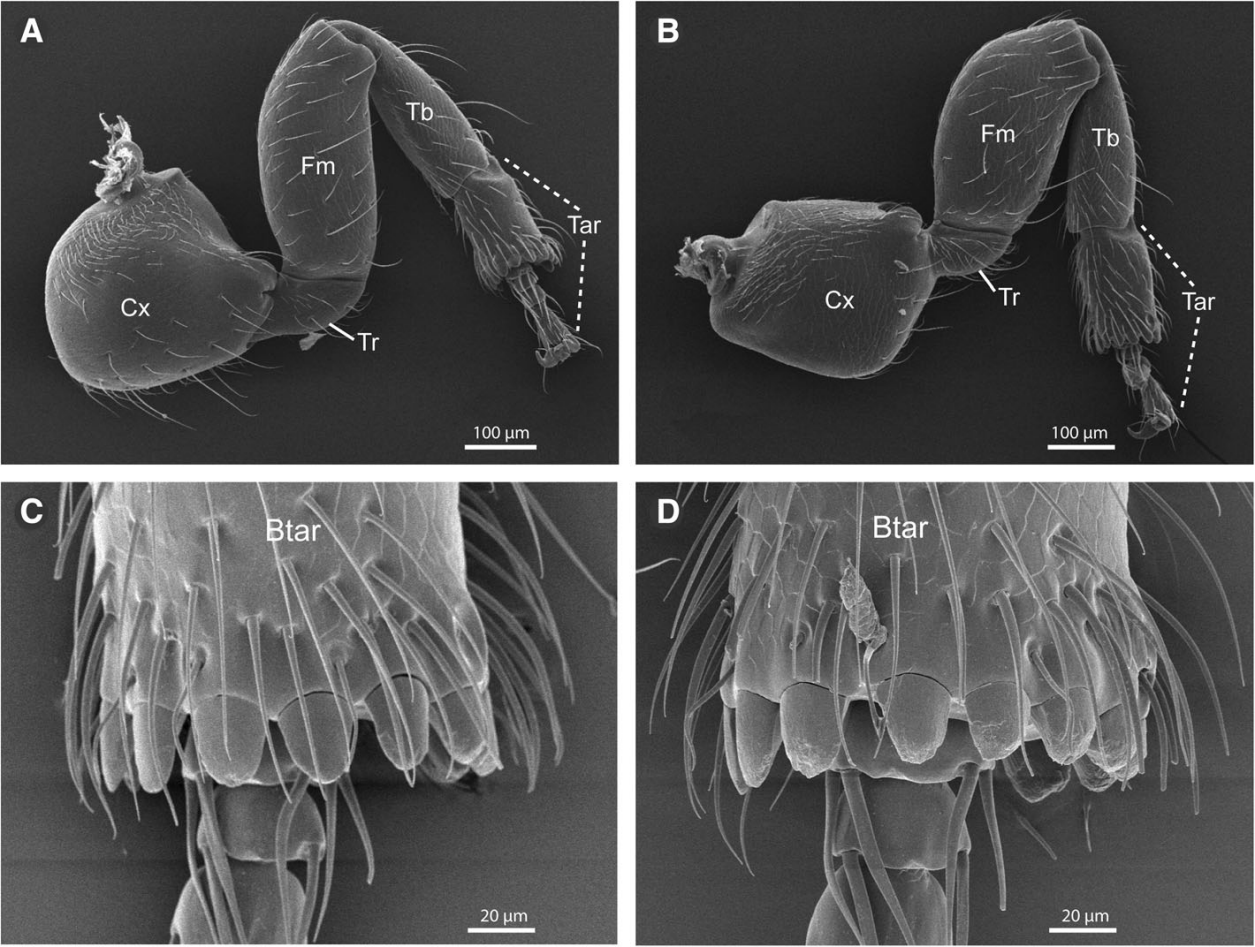
SEM images of mid and hind legs in *Melissotarsus* worker. Left (**a**) mid and (**b**) hind legs. Basitarsal peg-like setae of the midleg of a (**c**) young and (**d**) old worker. Cx, coxa; Tr, trochanter; Fm, femur; Tb, tibia; Tar, tarsus; Btar, basitarsus

*Melissotarsus* workers have hypertrophied mid- and hindcoxae that are almost as tall as the thorax (Fig. 3), unlike any other ants. These coxae are bulbous and subquadrate, not extending away from the sides of the body as in other ants. In addition, the trochanter in both these leg pairs bends 90° (Fig. 9a, b), directing the femur upward so that it is always in close contact with the lateral side of the body. The basitarsus of these legs is short and thick, with a diameter equal to that of the tibia with which it articulates broadly and with limited flexibility (assessed via direct dissections). This contrasts with the distal tarsomeres that are small and thin. These weak feet are offset by a row of stout peg-like setae at the distal end of each basitarsus (Fig. 9c, d). These tractor pegs show considerable wear in some individuals (Fig. 9d). Our micro-CT exploration of the legs (data not shown) revealed that the greatly enlarged coxae of mid and hind legs is packed with the muscles responsible for extension of the legs (i.e., moving the trochanter-femur complex away from the body).

## Discussion

### Head modifications as adaptations for chewing through live wood

The dorsoventrally enlarged head of *Melissotarsus* workers is packed with dorsal closer and ventral opener muscles. Ventral enlargement of the head provides space for large opener muscles hitherto unreported in other ants. In addition, the increased number of opener fibres in *Melissotarsus* connect to a spatula-shaped apodeme attached to the expanded outer process of mandibles. Such large sclerotized opener apodeme is not found in other insects [21, 22]. The unusually elaborate mandible opener mechanism in *Melissotarsus* apparently helps to disengage the mandibles from the elastic wood fibres while tunnelling – like a lumberjack removing his axe from a trunk after each stroke. Without this opening strength, workers would need to walk backwards to reopen their mandibles and chewing efficiency would decrease. It is the strong antagonistic contraction of opener and closer muscles that lies behind efficient tunnelling in live wood.

Not only are mandible muscles very large in *Melissotarsus* workers, but their output force is maximized through lever effects deriving from the broad base and prominent lateral (outer) process of mandibles. Insect mandibles have a triangular base in which two corners correspond to the mandible hinges and the third corner corresponds to the insertion of the closer muscle [7]. Accordingly, the closer muscles always pull the mandibles at a point much further away from the mandible hinge (the axis of rotation) than the opener muscles (Fig. 7). This is but a very basic lever system: by virtue of the insertion distance of the closer muscle from the fixed hinge, the input force of the closer muscles gets amplified, resulting in a greater output force in terms of biting. Moreover, the same holds for the lateral process where the opener muscle inserts: any elongation of this process will move the insertion of the opener muscle further away from the axis of rotation, increasing the lever effect and amplifying the input force. The broadening of the mandibular base and shorter length of *Melissotarsus* worker mandibles thus results in an increase in chewing force (both opening and closing actions). To our knowledge, such lever effects resulting from modified mandible geometry have not been described in other wood-chewing insects.

The pointy mandibles of young workers progressively abrade with age [6], evidence of intense strain while chewing. Together with zinc reinforced tips, widespread in insects (e.g. [23]), robust design makes *Melissotarsus* mandibles highly-suited for tunnelling in wood. Given the importance of mandibular sharpness for chewing, the special setae on the mandibles’ surfaces might act as a proprioceptor system to assess mandible wear: in young individuals the sharp tip is longer than any of the setae, so chewing will not exert any pressure on them; as the mandibles wear down, more and more of the setae will stick out and chewing will always mechanically stimulate them, signalling the worker that her mandibles are becoming less effective.

### Leg adaptations reflect a trade-off between walking and tunnelling

**Fig. 10:**
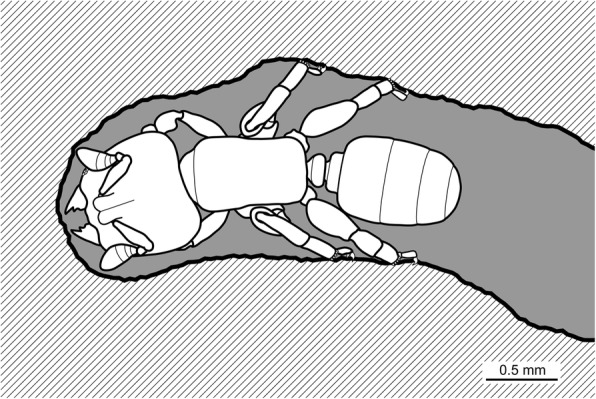
Representation of a *Melissotarsus* worker chewing a tunnel. Workers use their specialized mid and hind legs to brace themselves against the walls while tunnelling. Note how the basitarsi, and not the terminal tarsi, directly rest against the walls

*Melissotarsus* workers confine themselves inside their tunnels, which protect them against other ants and enemies. This irreversible commitment is evidenced by unique mid and hind legs adaptations, loss of the sting, and highly reduced eyes (about 12 ommatidia vs. 138 in queens). Eye reduction is a general characteristic of workers in hypogaeic ant species, while large eye size is the norm in arboreal ants [24], making *Melissotarsus* highly unusual. Giving up the ability to walk and forage outside allowed them to evolve novel specialisation of the legs: hypertrophied coxae with powerful muscles to push against the walls of their tunnels; 90° bent trochanters that help keep the legs close to the body; thick basitarsi armed with stout peg-like setae to increase traction. Most ant species walk inside tunnels constructed in the ground or plant matter, yet they do not exhibit such dramatic leg modifications. We suggest that the modified last two pairs of legs are not only appropriate for walking in tight spaces but also to rigidly anchor workers to the walls during tunnelling (Fig. 10), thus counteracting the strong chewing force of their equally specialized heads.

## Conclusions

*Melissotarsus* workers have developed a specialized morphology suited for their uniquely engineered ecological niche: an obligate mutualist partnership with diaspidid scale insects that feed inside tunnels chewed in healthy wood. Their head is large, packed with silk glands but also huge mandible closer and unusually large opener muscles. The insertion of these muscles and the shape of the mandibles itself maximize the force output for slow but powerful closing and opening motions. The remarkable design of mid and hind legs braces the body while chewing. These morphological adaptations for tunnelling evolved at the expense of normal walking and foraging, an unprecedented situation in ants. Polyphenism allows this extreme specialization of ant workers because the queen caste remains able to disperse by flight and walk on the outside of host trees during the first stages of colony foundation.

## Additional files

Additional file 2: **Table S1.** Summary of the micro-CT scan parameters for each specimen and body part analysed. (PDF 98 kb)
