# Supplementary material for: Skeletomuscular adaptations of head and legs of Melissotarsus ants for tunnelling through living wood
Source: Front Zool. 2018 Aug 14;15:30. doi: 10.1186/s12983-018-0277-6 (PMC6092875; doi:10.1186/s12983-018-0277-6)
Supplement: Supplementary file 2 — Table S1. Summary of the micro-CT scan parameters for each specimen and body part analysed. (PDF 98 kb) [file 12983_2018_277_MOESM2_ESM.pdf]

Supplementary material

Skeletomuscular adaptations of head and legs of *Melissotarsus* ants for tunnelling through living wood

Adam Khalife[1,2], Roberto A. Keller[3], Johan Billen[4], Francisco Hita Garcia[2], Evan P. Economo[2] & Christian Peeters[1,*]

[1] Sorbonne Université, CNRS, Institut d'Écologie et des Sciences de l'Environnement, Paris 75005, France

[2] Biodiversity and Biocomplexity Unit, Okinawa Institute of Science and Technology Graduate University, Onna, Okinawa, Japan

[3] MUHNAC/cE3c: Centre for Ecology, Evolution and Environmental Changes, Faculdade de Ciências, Universidade de Lisboa, Lisbon, Portugal

[4] Laboratory of Socioecology and Social Evolution, Zoological Institute, K.U. Leuven, Belgium

[*] Corresponding author: christian.peeters@upmc.fr

Table S1. Summary of the micro-CT scan parameters for each specimen and body part analysed.

Species	Caste	Body part	Specimen identifier	Voxel size (µm)	Exposure time (s)	Power (W)	Voltage (kV)	Amperage (µA)
Melissotarsus sp	Worker	Head	CASENT0790604	0.882	2.5	7	80	87
Melissotarsus sp	Worker	Thorax	CASENT0790604	1.126	2.5	7	80	88
Melissotarsus sp	Queen	Head	CASENT0790675	0.862	2.5	7	80	88
Melissotarsus sp	Queen	Thorax	CASENT0790605	1.218	2.5	7	80	88
Messor barbarus	Worker	Head	CASENT0790853	2.366	1	7	80	88
Messor barbarus	Worker	Thorax	CASENT0790853	2.662	3	4	50	81
